# A Cross-Study Transcriptional Analysis of Parkinson's Disease

**DOI:** 10.1371/journal.pone.0004955

**Published:** 2009-03-23

**Authors:** Greg T. Sutherland, Nicholas A. Matigian, Alistair M. Chalk, Matthew J. Anderson, Peter A. Silburn, Alan Mackay-Sim, Christine A. Wells, George D. Mellick

**Affiliations:** 1 National Centre for Adult Stem Cell Research, Eskitis Institute for Cell and Molecular Therapies, Griffith University, Brisbane, Queensland, Australia; 2 The School of Medicine, University of Queensland, Brisbane, Queensland, Australia; 3 Department of Neurology, Princess Alexandra Hospital, Brisbane, Queensland, Australia; 4 Department of Neurology, Royal Brisbane and Women's Hospital, Brisbane, Queensland, Australia; National Institutes of Health, United States of America

## Abstract

The study of Parkinson's disease (PD), like other complex neurodegenerative disorders, is limited by access to brain tissue from patients with a confirmed diagnosis. Alternatively the study of peripheral tissues may offer some insight into the molecular basis of disease susceptibility and progression, but this approach still relies on brain tissue to benchmark relevant molecular changes against. Several studies have reported whole-genome expression profiling in post-mortem brain but reported concordance between these analyses is lacking. Here we apply a standardised pathway analysis to seven independent case-control studies, and demonstrate increased concordance between data sets. Moreover data convergence increased when the analysis was limited to the five substantia nigra (SN) data sets; this highlighted the down regulation of dopamine receptor signaling and insulin-like growth factor 1 (IGF1) signaling pathways. We also show that case-control comparisons of affected post mortem brain tissue are more likely to reflect terminal cytoarchitectural differences rather than primary pathogenic mechanisms. The implementation of a correction factor for dopaminergic neuronal loss predictably resulted in the loss of significance of the dopamine signaling pathway while axon guidance pathways increased in significance. Interestingly the IGF1 signaling pathway was also over-represented when data from non-SN areas, unaffected or only terminally affected in PD, were considered. Our findings suggest that there is greater concordance in PD whole-genome expression profiling when standardised pathway membership rather than ranked gene list is used for comparison.

## Introduction

Parkinson's disease (PD, OMIM: #168600) is a uniquely human disease that is clinically characterised by cardinal motor symptoms such as postural instability, bradykinesia and resting tremor [1]. In the PD brain there is pathognomonic loss of more than 50% of the dopaminergic neurons in the substantia nigra pars compacta (SNc) located in the midbrain [2], [3]. However, the disease is also characterised by non-motor symptoms such as sleep disorders, depression, hyposmia and autonomic dysfunction [4]–[7]. Accordingly, pathology in PD is not just restricted to the SNc but also affects the olfactory pathway, spinal cord and dorsal cranial nuclei of the medulla [8]–[11].

High throughput discovery platforms, such as microarrays, that assume no *a priori* aetiological hypotheses, promise much in elucidating the pathogenesis of complex diseases such as PD. Moreover, one would hope that these microarray data would reveal clues previously inaccessible via other means. Several microarray-based studies have used human tissue to look for differentially expressed genes in Parkinson's disease [12]–[21]. The majority of these used post-mortem whole brain tissue from the substantia nigra (SN) [14]–[17] and although most authors emphasised differential expression in the ubiquitin-proteasome system or cellular energy pathways, their published gene lists appeared quite discordant. Others extended their studies to include pathologically normal brain regions [12], [18], [20], [21] and these highlighted other biological mechanisms such as G-Protein-coupled receptor signaling and transcriptional regulation.

One study assayed SNc dopaminergic neurons only, following isolation by laser capture microscopy (LCM) [13]. Here gender differences were more pronounced than PD versus control differences. Uniquely, one study compared the transcriptomes of whole blood samples [19] and reported expression differences in a number of unrelated genes.

Given an apparent lack of concordance in published data sets one might ask what relevance these transcriptional approaches can have to PD pathogenesis. Certainly the utilisation of post mortem brain tissue appears to represent the best opportunity for finding PD-specific changes in gene expression. Furthermore such ‘benchmarks’ facilitate the evaluation of clinical samples and model systems for their utility in PD research. However the approaches to generating and analysing microarray data are not standardised and therefore could account for much of the apparent discrepancy between reported gene lists.

Here we apply a common analytical approach to the available human transcriptomic data in an attempt to find greater data convergence and generate new insight into the pathways systematically altered during PD pathogenesis. We have also generated an online search tool and extend an invitation to other researchers to explore the data themselves http://ncascr.griffith.edu.au/pdreview/2008/.

## Results

### Demographics of microarray studies using PD case-control tissue

Ten published transcriptomic studies met the initial criterion of comparing primary tissues derived from PD patients and controls (Table 1). Of these, seven were included because they used the same common gene expression array. These seven studies provided 13 case-control data sets comparing tissue from 119 PD cases and 74 controls (Table 2). Three studies did not meet the inclusion criteria: (1) Grunblatt *et al.* [15] who used a Focus array (Affymetrix) with limited gene coverage; (2) Miller *et al* [22] who used the Codelink™ bioarray platform and (3) Bossers *et al.* [12] who used Agilent technology. We were also unable to source raw data from the study of Papapetropoulos and colleagues [18]. Although the raw data from these latter experiments was not included in our combined re-analysis, their published gene lists were used when initially evaluating data convergence between all studies.

**Table 1:** Summary of tissue used, size and array feature of published Parkinson's disease microarray publications.

First Author	PMID	Database Accession #	Tissue used in study	# of PD cases	# of controls	NPDC	Array Type	Summarisation & normalisation Methods	Statistical test used to determine differential expression
Grünblatt	15455214	-	SN,CB	7	7	-	HG-Focus	MAS5	Wilcoxon rank-sum test+fold-change cut-off
Hauser	15956162	-	SN	6	5	3 (2 PSP, 1 FTDP)	U133A	MAS5	2-sample t test
Vogt	16626704	-	OCTX, CB , PT	4	4	4 MSA	U133A	RMA	ANOVA
Zhang	15965975	-	SN ,PT, BA9	15	19	-	U133A	RMA	ANOVA+FDR
Miller	16143538	-	SN, STR	6&4	8&4	-	CodeLink	CodeLink	Student t-test
Moran	16344956	GSE8397	LSN, MSN, SFG	15&9&5	8&7&3	-	U133A+B	GC-RMA+MAS5+PLIER	two-class unpaired+FDR
Lesnick	17571925	GSE7621	SN	16	9	-	U133 plus 2.0	MAS5	ANOVA
Scherzer	17215369	GSE6613	whole blood	50	22	AD, PSP, MSA, CBD, ET)	U133A	MAS5	LOOCV
Castelvetri	17412603	E-MEXP-1416	LCM DA-SN	8	8	-	X3P	GC-RMA	SAM
Bossers	18462474	-	SN, PT, CN	3	4	1 PD/DEM	Agilent 22K	Loess	Linear regression with FDR

**PMID -** PubMed ID, **SN** - Substantia nigra, **LSN** - Lateral SN, **MSN-** Medial SN, **SFG-** Superior Frontal Gyrus, **BA9-** Brodmann Area 9, **PT-** Putatmen, **LCM DA-SN** - Laser Captured Dopaminergic SN, **OCTX-** Occipital Cortex, **CB-** Cerebellum, **CN** - Caudate nucleus, **NPDC** -Non Parkinson Disease controls, **PSP -** Progressive Supranuclear Palsy, **FTDP** - Frontotemporal Dementia, **MSA -** Multiple System Atrophy, **CBD** - Corticobasal degeneration, **PD/DEM** – PD with dementia, **ET - ANOVA** -Analysis of Variance, **FDR** - False Discovery Rate, **LOOCV -** Leave-One-Out Cross-Validation, **SAM -** Significant Analysis of Microarray.

**Table 2:** Summary of each data set used in the re-analysis and the number of differentially expressed probes before and after neuronal correction.

Studies	Number of PD patients used in microarray analysis	Number of controls used in microarray analysis	RNA tissue source	Number of differentially expressed probes ≤0.01	Number of differentially expressed probes ≤0.01 after neuronal correction*
**Substantia nigra Data Sets**					
Hauser	6	5	SN	159	152
Zhang	11	18	SN	1014	951
Moran	15	6	LSN	1975	1779
Moran	15	6	MSN	2149	1924
Lesnick	16	9	SN	2030	1993
**Other Non-SN Brain Regions Data Sets**					
Zhang	14	19	BA9	2373	-
Zhang	15	15	PT	197	-
Moran	15	6	SFG	598	-
Vogt	4	4	OCTX	1727	-
Vogt	3	3	PT	155	-
Vogt	4	4	CB	174	-
**Other Data Sets**					
Castelvetri	8	8	LCM DA-SN	491	-
Scherzer	55	22	Blood	208	-

* no probes were removed from the non-SN brain regions data sets.

### Lack of convergence of pathways between studies from published ranked gene lists

We postulated, as others had done previously, that genes and pathways that appear consistently as differentially expressed in multiple studies and different source tissues are likely to be important in PD [20]. To look for such convergence we compared the published lists of differentially expressed genes using the Ingenuity Pathways Analysis (IPA) package. There was little overlap between published ranked gene lists and there were no genes consistently identified in more than three datasets (Table S1 ). We then asked whether we would see greater convergence using pathway analysis of the originally reported ranked gene lists. The ERK/MAPK was the most highly represented pathway, although it was over-represented in only four of the 11 data sets (Table 3).

**Table 3:** Summary of over-represented IPA pathways from the published array data.

IPA Pathway category	Number of studies with over-represented pathways at ≤0.05 in all published lists (11)
ERK/MAPK Signaling	4
G-Protein Coupled Receptor Signaling	3
Huntington's Disease Signaling	3
α-Adrenergic Signaling	3
Synaptic Long Term Potentiation	3
PPARα/RXRα Activation	3

### Application of common data analysis methodology

Our common analysis method was applied to the 13 datasets (from seven studies) that met our platform inclusion criteria, and new ranked probe lists were produced. These are listed in Table S2, while the number of differentially-expressed probes is shown in Table 2. Additionally these data can be viewed online http://ncascr.griffith.edu.au/pdreview/2008/. This improved the convergence of the genes between the datasets with 20 genes now consistently differently regulated across six of 13 datasets (data not shown). Ranked IPA pathways from these gene lists can be found in Table S3.

### Pathway analysis of SN data sets reveals a common dysregulation of dopamine signaling

Dopaminergic neuron loss in the SNc is the prominent neuropathological entity in PD so we initially focused on the SN data sets for their convergence and reproducibility. Over-representation of the dopamine receptor signaling pathway was consistently and significantly observed in all SN data sets (p-values >0.003–0.026) suggesting that not only can PD-related pathways be dissected out of complex transcriptomic data but that these changes are robustly reproducible between comparable studies (Table 4 and Table S2 A–E for full ranked pathway lists).

**Table 4:** Summary of over-represented IPA pathways in the SN data sets before and after neuronal correction.

IPA Pathway category	Number of studies with over-represented pathways at ≤0.05 in SN data sets (n = 5)	Number of studies with over-represented pathways at ≤0.05 in SN data sets (n = 5) after neuronal correction
Dopamine Receptor Signaling	5	1
IGF-1 Signaling	3	2
PTEN Signaling	3	3
JAK/Stat Signaling	3	3
Glucocorticoid Receptor Signaling	3	3
Huntington's Disease Signaling	3	3
PPAR Signaling	3	3
Ephrin Receptor Signaling	2	4
VEGF Signaling	2	2
Axonal Guidance Signaling	2	3
PI3K/AKT Signaling	2	2
Insulin Receptor Signaling	2	2
BMP signaling pathway	2	2
Synaptic Long Term Depression	2	2
Synaptic Long Term Potentiation	2	0
PDGF Signaling	2	1
B Cell Receptor Signaling	2	2
Lysine Degradation	2	2
Estrogen Receptor Signaling	2	2
G-Protein Coupled Receptor Signaling	2	0
Inositol Phosphate Metabolism	2	1
IL-2 Signaling	2	1

Neuronal loss in PD is more severe in the lateral SN compared to the medial SN [23]–[25] while areas such as the superior frontal gyrus (SFG) are largely unaffected. A comparison of these three anatomical areas from the data in Moran *et al.* (LSN v MSN v SFG) showed a direct correlation between fold changes in genes of dopamine receptor signaling pathway and the severity of PD neuronal loss [14]. Therefore, the prominence of this pathway appeared to represent the disparate numbers of residual dopaminergic neurons in PD compared to control brains.

### Correcting for dopaminergic neuronal loss changes ranked pathways

In order to bias our analysis towards underlying pathogenic mechanisms rather than terminal pathology, we devised a correction paradigm based on Moran's observations on neuronal loss. Our rationale is described in detail in Table S4. 217 genes with fold changes LSN>MSN>SFG from the Moran *et al.* data sets were defined as potentially “neuronal-loss-associated”, as these were the genes whose differential expression most likely resulted from relative loss of dopaminergic neurons from the brain regions sampled, rather than transcriptomic differences in residual cells. However eight of these genes could be shown to be differentially expressed in residual dopaminergic neurons (Cantuti-Castelvetri *et al.* study)[13] and so were retained in the analysis. The removal of 209 genes (Table S5) from the SN ranked lists resulted in two alternative pathways gaining prominence: ephrin receptor signaling (p-values <0.003–0.04) and the axonal guidance pathway (p-values <0.004–0.049) (Table 4 and Table S6 A–E for full ranked pathway lists).

### Assessment of glial contribution to the neuronal-loss corrected datasets

Given the loss of dopaminergic cells in the PD SN the major contribution to the expression profile in the SN PD samples would presumably now come from the non-dopaminergic cells. Furthermore neuropathology in the PD SN is characterised by a reactive gliosis or “glial inflammation” (reviewed by Orr *et al.*, 2002) [26]. Therefore glial markers and in particular reactive microglia markers such as CD68 and ICAM-1 might be expected to be upregulated in the SN data sets [26], [27]. However these genes and those of glial markers in general were largely indifferent between PD and controls. This potential anomaly is illustrated further using a selection of PD-related glial markers (Table S7).

### Analysis of non-SN brain tissue highlights growth factor signaling pathways

We also analysed non-SN tissues as they are not subject to cytoarchitectural changes seen in the SN or are only affected late in the disease. Five IPA pathways were overrepresented in three out of five non-SN data sets (Table 5). Of these, IGF-1 and VEGF signaling were also found to be dysregulated in two of the neuronal loss-corrected SN datasets. Whereas, the four pathways (PTEN signaling, JAK/STAT signaling, ephrin receptor signaling, axonal guidance signaling) found to be significance in three or more of the corrected SN datasets were only significance in two of the non-SN datasets.

**Table 5:** Comparison of over-represented IPA pathways in non-SN data sets and neuronal-loss corrected SN data sets.

IPA Pathway category	Number of studies with over-represented pathways at ≤0.05 in non-SN data sets (n = 6)	Number of studies with over-represented pathways at ≤0.05 in SN data sets (n = 5) after neuronal correction
IGF-1 Signaling	3	2
VEGF Signaling	3	2
Synaptic Long Term Potentiation	3	0
Calcium Signaling	3	0
ERK/MAPK Signaling	3	0
PTEN Signaling	2	3
JAK/Stat Signaling	2	3
Ephrin Receptor Signaling	2	4
Axonal Guidance Signaling	2	3

### Differential expression in whole blood

Finally, given its clinical accessibility, we also re-analysed a whole blood dataset [19]. Our analysis revealed that the inositol phosphate metabolism and VEGF signaling pathways were the most differentially expressed in this dataset. The prominence of these pathways was quite distinct from SN tissues but commonality with non-SN tissues was observed with the VEGF signaling pathway (Table S2 L).

## Discussion

Microarrays promise much in elucidating the pathogenesis of complex diseases such as PD but the lack of concordance in published data sets to date certainly questions their relevance. Here we have shown that a standardised approach to analysing PD-related microarray data can account for a considerable proportion of the discordance. We used a common analytical approach which improved data convergence and uncovered new leads for PD pathogenesis. We also recognised a potential anatomical bias in the datasets derived from brain regions with high neuronal loss. Our approach therefore provided an improved comparative analysis between existing datasets and further considered ‘tissue-of-origin’ effects.

### Pathway analysis may uncover concordance between datasets not found in gene lists

Complex phenotypes, by their very nature, are aetiologically heterogeneous. This implies that single gene signatures may not be shared by all affected individuals. However, the identification of particularly relevant genetic pathways, have a higher probability of being revealed as convergent across multiple individuals and multiple studies than individual genes *per se*. Moreover, the differences reflected in a pathway or network of genes may be robust enough to overcome the effects of experimental noise and inter-study variability prone to bias single gene expression values. Therefore, this approach results in an increase in sensitivity to detect interesting and novel patterns in gene expression between multiple samples of cases and controls.

### Common data analysis highlights dopamine signaling pathway in SN

Context is very important in gene expression studies and as expected the analysis of SN tissue-derived data sets further improved our concordance and highlighted the ‘dopamine receptor signaling’ pathway. However rather than representing a primary pathogenic effect the extensive down regulation of genes such as DOPA decarboxylase (*DDC*), dopamine receptor 2 (*DRD2*), dopamine transporter (*SLC6A3* or *DAT*) and tyrosine hydroxylase (*TH*) was probably entirely due to a disproportionate number of SN dopaminergic neurons between cases and controls. On a positive note, the microarray data was providing accurate molecular fingerprints of the comparative tissue being examined; a modern pixelated analogy to the histological section. However the relative neuronal loss in the SN also reduced our signal to noise ratio for pathogenic relevance.

Accordingly we pursued two alternative approaches to maximise potentially useful information on the underlying biological processes. First, the implementation of a correction factor for dopaminergic neuronal loss in the SN data sets and second, the analysis of non-SN or unaffected tissues for data convergence. The purpose of the correction factor was not to magically recreate the early disease landscape but to remove ‘red herrings’ that solely reflected the relative numbers of neurons between cases and controls. The retention of genes differentially regulated in the residual dopaminergic neuron data set should have improved the overall specificity of this approach.

Following our ‘neuronal loss’ correction, two alternative pathways gained prominence: ephrin receptor signaling and the axonal guidance pathway. The latter is consistent with the findings of recent studies [12], [28] including one that combined gene expression data with genotype data from two genome-wide association studies [28]. There is actually considerable overlap between the axon guidance and ephrin signaling pathways with ephrins along with netrins, slits and semaphorins being the main families of guidance molecules in the developing nervous system [29]. It remains to be clarified whether the differential expression of axon guidance genes in PD represent neurodevelopmental manifestations, compensatory attempts at rewiring, or dysregulated expression patterns induced by a devastated environment.

As discussed above microarray data is very powerful in illustrating cytoarchitectural differences between cases and controls such as dopaminergic neuron loss. Given the considerable literature supporting the involvement of glia in PD pathogenesis [26], [27], [30], [31] we might have expected glial markers to inversely differentiated in the SN data sets. The absence of significant fold changes in activated microglial markers in particular argues against a substantial glial component to neurodegeneration in the terminal PD brain.

### Growth factor signaling pathways are prominent in non-SN tissues

The distinct gene expression pattern of brain areas that are not overtly affected by PD pathology may be less confounded than the SN with respect to the cell death associated with PD. It could be argued that the transcriptomes of unaffected tissues might be too divergent from those of predilection sites such as the SN, such that they provide very little informative data. Our analysis, which has highlighted consistent differences in growth-factor signaling in non-SN datasets, argues that areas affected late in the disease, such as the prefrontal cortex [8] could at the time of post mortem exhibit similar mechanisms of degeneration as initially occurred in the SN. Our approach highlights the differential expression of IGF-1 and VEGF signaling pathways. Importantly the IGF1 signaling pathway was also over-represented in two ‘corrected’ SN datasets (Table 5).

This pathway has been largely unexamined for associations with PD although IGF1 signaling is reported to have neuroprotective effects on dopaminergic neurons [32], [33] and it has recently been suggested that excessive IGF1 signaling accelerates ageing through deleteriously effects on protective mechanisms against proteotoxicity such as Lewy body formation in PD [34]. Furthermore we have recently showed that a polymorphism in the 3′ untranslated region of the IGF2 gene, a homologue of *IGF1* was protective against PD [35].

Similarly VEGF is known to promote the growth and survival of dopaminergic neurons [36]–[38] Interestingly both IGF1 and insulin enhance VEGF expression *in vitro* [39], [40] providing a plausible mechanism that might underlie the co-prominence of these signaling pathways in our re-analysis.

### Challenges and Future Directions

Case-control expression analysis in a degenerative disease like PD poses difficult issues when attempting to uncover pathways contributing to disease initiation. It would be advantageous to target tissues that express the proteins that are fundamental to the disease process and are different in individuals who are at risk of the disease. At the same time we need to account for any influences of the pathological process on these profiles. Microarray data of predilection sites such as the SN illustrates cytoarchitectural differences between cases and controls but to understand some of the early pathogenic processes, we would ideally want to assay a brain region very similar to SN but that is only belatedly affected.

An additional consideration is the ability of the pathway approach, used in our analyses, to provide adequate specificity for PD over other neurodegenerative conditions. This issue remains to be clarified, and requires further investigation. It is important to recognise that there may be genetic expression patterns common to neurodegenerative diseases, generally. These may reflect common pathological changes (such as cell death, markers of oxidative stress or neuro-inflammation etc) or shared risk factors influencing neurodegeneration.

There are still inherent difficulties in obtaining reproducible gene expression data from post mortem brain, even if an optimal region of the brain could be assayed [41]–[44]. Furthermore this information can only be used retrospectively for the potential benefit of future PD patients. Therefore there is considerable interest in developing strategies to obtain human RNA from more accessible sources such as blood or neuronal-like cell lines. The finding of down regulation of α-synuclein in microarray analysis of whole blood samples from PD patients versus controls is exciting because it implies that PD-specific changes can be found *ante mortem* in readily accessible tissues [19]. It is also of note that these tissues also revealed differential expression of the VEGF signaling pathway (observed in other non-SN tissue samples). However, there is a real risk that peripheral tissues, such as whole blood examined here, may express few proteins fundamental to the disease process and therefore be of limited ability to demonstrate case - control differences relevant to nervous system disorders [45]. The lack of available gene expression data from multiple tissues in PD patients at various stages of the disease prevents such an analysis but highlights the need for ongoing research efforts in this area.

Interestingly one peripherally accessible neural tissue, the olfactory mucosa, has been used to demonstrate significant differences in functional assays and gene expression between schizophrenics, bipolar affective disorder and controls [46]. Such cells from PD patients and controls may yet provide an opportunity to interrogate neuronal mechanisms without relying on post mortem tissue [47].

In this paper we have presented a summary of the available microarray data from PD case-control studies and have suggested some potential strategies for uncovering primary pathogenic mechanisms. For others who wish to use and explore these data we have constructed an online database which enables rapid evaluation on a single gene or pathway basis.

## Materials and Methods

We conducted literature searches in National Center for Biotechnology Information (NCBI) PubMed and dataset searches in NCBI gene expression omnibus and ArrayExpress (EBI) [48], [49] to identify all reported microarray studies that explored differential gene expression in Parkinson disease. Studies satisfied the inclusion criteria if they: 1) compared tissues from PD patients and controls; 2) assessed transcripts on a genome-wide basis; and 3) used Affymetrix gene expression arrays. For the selected studies we obtained raw microarray data (CEL files) from public microarrays repositories [13], [14], [19] or from the study authors [16, Vogt, 2006 #1529,21,]. In one case the data was publicly available from the follow up study [28] rather than the primary study [18].

### Meta-analysis data summarisation, normalisation and analysis of variance

All studies used Affymetrix arrays, the probes on the arrays and the experimentally chosen fluorescence thresholds varied. Consequently, the data could not be simply combined without avoiding study bias and the effects of probe-level sequence information [50]. To overcome this problem, the raw data (as CEL files) for each dataset were imported individually into GeneSpring® ™ 7.3.1(Agilent) and the probes sets were summarised and normalised by the Robust Multichip Average (RMA) algorithm[51]. Some studies included non-PD disease controls but our analysis was performed on PD and control patients only (Table 1). For studies which used multiple brain regions, each area was treated as a separate data set. Differentially expressed genes between PD and controls were determined by an analysis of variances (ANOVA) using a Welch t-test with a p-value cut-off of ≤0.01.

### Pathway over-representation analysis

The ranked genes lists for each study were assessed by integrating the data at a pathway level. Each ranked list was imported into the Ingenuity Pathways Analysis 6.3 (IPA, from Ingenuity® Systems, www.ingenuity.com) which incorporates an extensive literature-derived knowledge base from which to assign pathway affiliation. The significance value for pathway over-representation was calculated using a right-tailed Fisher's exact test. Each pathway was ranked by assessing the number of studies that were statistically over-represented (p-value ≤0.05). This pathway over-representation ranking was performed individually on data sets utilising substantia nigra (SN) (Hauser, Moran LSN+MSN, Zhang, Lesnick)[14], [16], [21], [28] and non-SN tissues (Zhang-Putamen, Zhang-BA9, Moran-SFG, Vogt-OCT, Vogt-Putamen, Vogt-CB)[14], [20], [21]. Additionally this analysis was performed independently on data sets derived from whole blood (Scherzer)[19] and dopaminergic neurons (Cantuti-Castelvetri)[13].

### Correction for dopaminergic neuronal loss

The substantia nigra pars compacta of PD patients is characterised by the loss of neuromelanin-containing dopaminergic neurons [2], [3]. Furthermore neurons are lost in a particular pattern; severity decreasing from ventrolateral to dorsomedial [23]–[25]. In an attempt to correct for the effects on expression arising directly from the neuronal loss associated with PD, we devised the following correction paradigm. We first used data from the Moran *et al.* study[14] which compared gene expression profiles in three brain regions, lateral SN (LSN), medial SN (MSN) and superior frontal gyrus (SFG), from the same patients. The actual neuronal loss in PD is known to be greater in LSN compared to MSN, with the SFG relatively spared. Therefore significant probes (p≤0.01) with fold changes in LSN>MSN>SFG were defined as potentially “neuronal-loss-associated”. For example the tyrosine hydroxylase (TH) gene showed a fold change pattern of −14.5 (LSN), −4.9 (MSN) and no change (SFG). Any probes which were defined as “neuronal-loss-associated” and were not differentially expressed in residual laser-captured dopaminergic neurons (Cantuti-Castelvetri *et al.* study)[13] were removed from the subsequent analyses of all SN data sets.

### Online Database

The differentially expressed gene list generated for each study by this re-analysis can be found with their respective p-values and fold changes can be found at http://ncascr.griffith.edu.au/pdreview/2008/. A search can be performed individually using Entrez gene ID, gene symbol, or collectively by publicly available lists/pathways.

### Accession Numbers

The National Center for Biotechnology Information Entrez Gene website (http://www.ncbi.nlm.nih.gov/sites/entrez?dbgene) accession numbers (GeneIDs) for the genes named in the paper include: *DDC*(1644), *DRD2*(1813), *SLC6A3*(6531), *TH*(7054), *IGF2*(3481).

## Supporting Information

Table S1 Comparison of overlap in genes between PD-related transcriptomic studies. The enclosed table illustrates the increase in data convergence between PD-related transcriptomic studies following the implementation of our common analysis methodology.
(0.02 MB PDF)

Table S2 The differentially expressed probes generated by common analysis for each study. Ranked probe lists for each study generated by common analysis method with fold change and p-value.
(1.08 MB XLS)

Table S3 Over-represented pathway categories from IPA analysis. Over-represented pathway categories from IPA analysis of the differentally expressed probes of PD patients compared to controls: A- SN - Hauser study; B- SN - Zhang study; C- lateral SN - Moran study; D- medial SN - Moran study; E- SN - Lesnick study; F- Brodmann Area 9 - Zhang study; G- Putamen - Zhang study; H- Superior Frontal Gyrus - Moran study; I- Occipital Cortex - Vogt study; J- Putamen - Vogt study; K- Cerebellum - Vogt study; L- Whole Blood - Scherzer study; M- Laser Captured SN dopaminergic neurons - Castelvetri study.
(0.07 MB XLS)

Table S4 Selection of neuronal loss-associated genes. A work flow diagram and hypothetical examples illustrate the selection of neuronal loss-associated genes that were removed from the SN datasets prior to pathway analysis.
(0.12 MB PDF)

Table S5 ‘Neuronal-loss’ associated genes removed by correction paradigm. Identified probes with fold change's for the three brain regions in the Moran study that followed the pattern, LSN>MSN>SFG.
(0.05 MB XLS)

Table S6 Identified probes with fold change's for the three brain regions in the Moran study that followed the pattern, LSN>MSN>SFG. Over-represented pathway categories from IPA analysis after neuronal correction of the differentally expressed probes from SN tissue: A- Hauser study; B- Zhang study; C- lateral SN; Moran study; D- medial SN Moran study; E- Lesnick study.
(0.04 MB XLS)

Table S7 Fold changes in PD-related glial markers. The lack of differential expression of PD-related glial markers is illustrated in the enclosed table.
(0.07 MB PDF)
