# Supplementary material for: A Cross-Study Transcriptional Analysis of Parkinson's Disease
Source: PLoS One. 2009 Mar 23;4(3):e4955. doi: 10.1371/journal.pone.0004955 (PMC2654916; doi:10.1371/journal.pone.0004955)
Supplement: Table S1 — Comparison of overlap in genes between PD-related transcriptomic studies. The enclosed table illustrates the increase in data convergence between PD-related transcriptomic studies following the implementation of our common analysis methodology. (0.02 MB PDF) [file pone.0004955.s001.pdf]

Table S1. Comparison of overlap in genes between PD-related transcriptomic studies

The following table illustrates the increase in data convergence between PD-related transcriptomic studies following the implementation of our common analysis methodology

	Number of genes in common	
# of Studies	From the 11 published gene lists	From the 13 gene lists after common analysis
8	0	0
7	0	2
6	0	20
5	0	53
4	0	170
3	8	565
2	54	1389
1	693	3385
