# Supplementary material for: A Cross-Study Transcriptional Analysis of Parkinson's Disease
Source: PLoS One. 2009 Mar 23;4(3):e4955. doi: 10.1371/journal.pone.0004955 (PMC2654916; doi:10.1371/journal.pone.0004955)

Table S4. Selection of neuronal loss-associated genes

We selected genes from the three Moran *et al.* datasets with statistically significant differences in expression between PD and controls, and subsequently remove those genes that fulfil the criteria: LSN>MSN>SFG [14]. This equates to regions where the magnitude of dopaminergic neuronal loss is likely to range from severely affected (LSN), moderately affected (MSN), to unaffected (SFG).

LSN	MSN	SFG	Neuronal loss associated gene
-4	-2	ND(=0)	Yes
ND(=0)	ND(=0)	-1.5	No
ND(=0)	-2	-0.5	No
-4	-2	-1.5	Yes
-4	ND(=0)	-2	No
-4	-2	-1.5	Yes
-2	-2	ND(=0)	No
-2	2	-1.5	No
4	2	1	Yes
2	4	6	No

ND= Not significantly different between PD and Controls.

Schematic of workflow for selecting neuronal loss-associated genes.

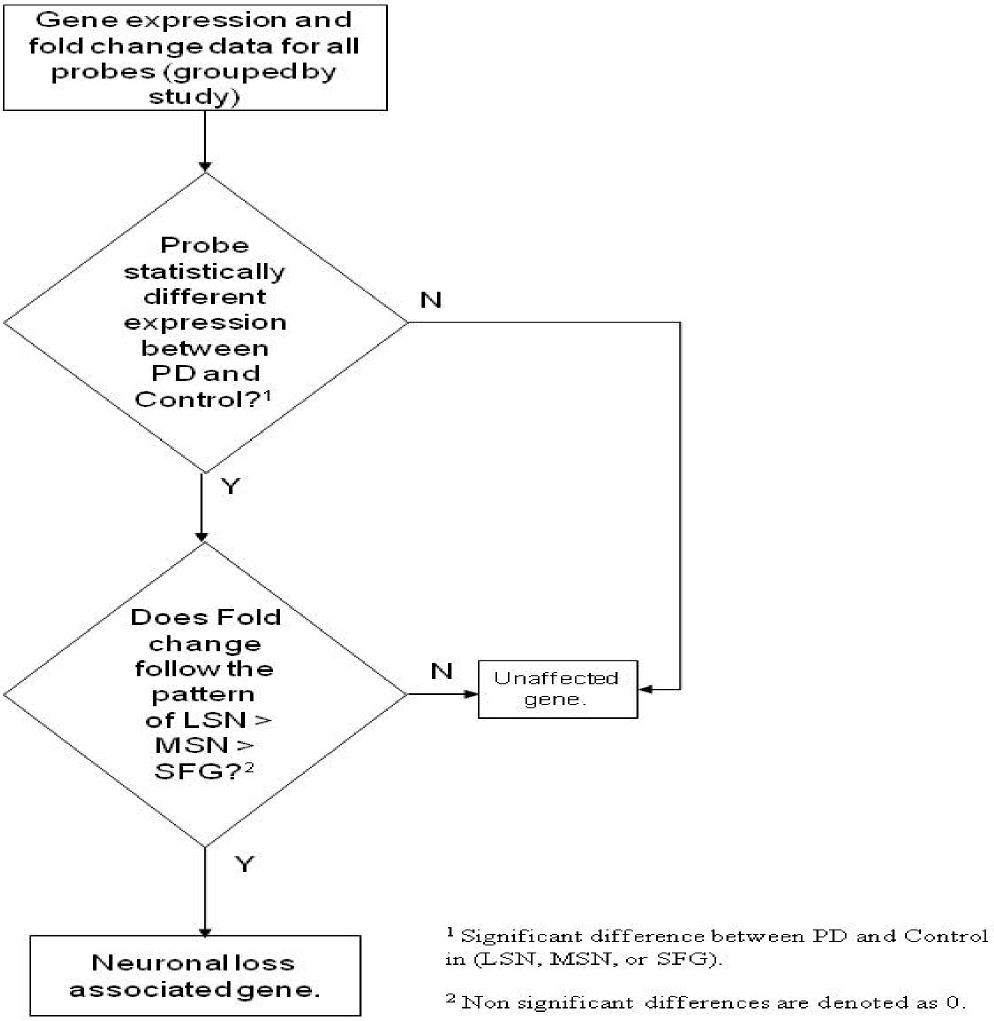

Supplement: Table S4 — Selection of neuronal loss-associated genes. A work flow diagram and hypothetical examples illustrate the selection of neuronal loss-associated genes that were removed from the SN datasets prior to pathway analysis. (0.12 MB PDF) [file pone.0004955.s004.pdf]
