# Supplementary material for: A Cross-Study Transcriptional Analysis of Parkinson's Disease
Source: PLoS One. 2009 Mar 23;4(3):e4955. doi: 10.1371/journal.pone.0004955 (PMC2654916; doi:10.1371/journal.pone.0004955)
Supplement: Table S7 — Fold changes in PD-related glial markers. The lack of differential expression of PD-related glial markers is illustrated in the enclosed table. (0.07 MB PDF) [file pone.0004955.s007.pdf]

Table S7. Fold changes in PD-related glial markers

Symbol	Gene Name[#]	LSN Duke FC*	MSN Duke FC*	SFG Duke FC*	SN Hauser FC*	SN Zhang FC*	SN Lesnick FC*
**Astroglia**		-	-	-	-	-	-
GFAP	glial fibrillary acidic protein	-	-	-	-	-	-
S100B	S100 calcium binding protein B	-	-	-	-	-	-
SLC1A3	solute carrier family 1 (glial high affinity glutamate transporter), member 3	-	-	-	-	-	-
SLC1A2	solute carrier family 1 (glial high affinity glutamate transporter), member 2	-	-	-	-	-	-
GLUL	glutamate-ammonia ligase (glutamine synthetase)	-	-	-	-	1.26	-
GDNF	glial cell derived neurotrophic factor	-	-	-	-	-	1.2
BDNF	brain-derived neurotrophic factor	-	-	-	-	1.26	-
NFE2L2	nuclear factor (erythroid-derived 2)-like 2	-	-	-	-	-	-
F2R	coagulation factor II (thrombin) receptor (PAR-1)	-	-	-	-	-	-
ARMET	arginine-rich, mutated in early stage tumors (MANF)	-	-	-	-	-	-
**Microglia**		-	-	-	-	-	-
ITGAM	integrin, alpha M (complement component 3 receptor 3 subunit)	-	-	-	-	-	-
CD68	CD68 molecule	-	-	-	-	-	-
CD40	CD40 molecule, TNF receptor superfamily member 5	-	-	-	-	-	-
CD80	CD80 molecule	-	-	-	-	-	-
CD86	CD86 molecule	-	-	-	-	-	-
ICAM1	intercellular adhesion molecule 1	-	-	-	-	-	-
**Oligodendrocytes**		-	-	-	-	-	-
MAP4	microtubule-associated protein 4	-	1.28	-	-	-	-

Markers from Orr et al., 2002 and McGeer and McGeer, 2008 [26,27]

* FC = fold change of significantly different genes (p-value < 0.01)
